# Age friendly states as a linchpin of age friendly ecosystems

**DOI:** 10.1093/geront/gnag063

**Published:** 2026-04-28

**Authors:** Claire Wickersham, Caitlin Coyle

**Affiliations:** LeadingAge LTSS Center, University of Massachusetts Boston, Boston, Massachusetts, United States; The Center for Social and Demographic Research on Aging, University of Massachusetts Boston, Boston, Massachusetts, United States

**Keywords:** Age-friendly state, Multisector plan on aging, Master plan on aging, Multisectoral plan on aging

## Abstract

States across the United States are advancing the age-friendly movement by joining networks that promote age-friendly communities. Although much of the existing scholarship has focused on local initiatives, far less attention has been paid to the role of states in sustaining and scaling age-friendly change. Age-Friendly States build on local momentum to drive systems-level changes that create healthier, more inclusive environments for older adults. This forum examines the role of states in shaping sustainable aging policy and practice. An environmental scan of age-friendly state plans and multisector plans on aging was conducted to document and examine how states design and implement approaches to healthy aging and to identify promising strategies for systems change across public sectors. Nineteen states met the inclusion criteria: 12 had AARP Age-Friendly State designations, eight had Multisector Plans for Aging in development or implementation, and four had Multisector Plans for Aging supported by legislation or executive orders. Findings from this analysis were synthesized to surface transferable models, enabling conditions, and actionable lessons that can inform future state and local efforts to embed age-friendly principles across systems to support long-term, equitable change. The authors argue that states involvement is a crucial lynchpin in advancing age-friendly ecosystems.

The age-friendly movement emerged in response to a global recognition of the importance of creating livable communities for aging populations. Initially championed by the World Health Organization’s Age-Friendly Cities and Communities initiative, the framework encourages communities to adapt infrastructure, services, and policies that promote active aging and social inclusion (WHO, 2007). Unlike local governments, states possess the statutory authority, financing mechanisms, and cross-agency coordination capacity necessary to translate age-friendly principles into durable systems change. In the United States, states are uniquely positioned to scale these efforts, acting as intermediaries between federal and local governments. They shape environments through legislation, resource allocation, and interagency coordination (Greenfield & Pestine-Stevens, 2022). As demographic shifts intensify, the imperative for state leadership in fostering inclusive, age-friendly societies has become increasingly urgent.

Recent scholarship emphasizes that age-friendly efforts are most impactful when viewed as part of a broader *age-friendly ecosystem—*a coordinated system of care, services, and supports that spans government agencies, private industry, and community partners (Graham, 2024). Multisector Plans for Aging (MPAs), also known as Master Plans on Aging, are now underway in more than half of U.S. states and exemplify this systems-based approach by establishing shared priorities and aligning initiatives under a unified, state-led blueprint (Graham & Hoffmaster, 2022). Although the AARP Age-Friendly State framework has been instrumental in catalyzing community-level participation, MPAs differ in that they are formal state-level planning processes with direct legislative authority, built-in accountability mechanisms, and, in some cases, dedicated funding streams (Berkowsky et al., 2024; Rauscher et al., 2024). Together, these frameworks highlight both the opportunities and the challenges for scaling age-friendly change at the state level.

The AARP Network of Age-Friendly States and Communities has catalyzed a national movement, promoting planning and collaboration at both the local and state levels. States enrolling in the AARP Network must submit a formal commitment from the Governor, commit to improving key domains, including housing, transportation, health services, and civic engagement, and submit periodic progress reports to maintain designation status (AARP, n.d.). Participation in the network is voluntary and facilitates knowledge sharing and accountability, encouraging the integration of age-friendly principles into policy development and implementation.

Alongside the AARP effort, the national MPA movement has introduced a complementary pathway for states to embed aging-related goals across various sectors. MPAs are typically state-led and developed through cross-agency collaboration, stakeholder engagement, and a commitment to data-informed planning and management (Rauscher et al., 2024). They are “living documents,” adaptable to emerging needs, and are increasingly formalized through legislation or executive orders (Rauscher et al., 2024).

The age-friendly state model acknowledges the necessity for sustained, coordinated efforts across multiple systems. It facilitates alignment between grassroots initiatives and statewide frameworks, ensuring consistent goals, metrics, and resource allocation. By standardizing age-friendly policies, promoting innovation, and fostering partnerships among aging, health, housing, and transportation systems, states can reduce barriers to local implementation and enhance the overall quality of life for older adults (Greenfield & Pope, 2025). An age-friendly state is a linchpin in age-friendly ecosystems because it establishes the policy authority, cross-agency alignment, and financing structures necessary to scale and sustain local age-friendly efforts, translating community-level innovation into durable systems change that shapes how transportation, housing, health, and social services operate across the life course.

## Environmental scan of age friendly states

This study reviewed 18 states and one U.S. territory with active or emergent age-friendly initiatives. States were selected based on established criteria, such as enrollment in the AARP Age-Friendly Network, the presence of a formal MPA, or documented policy actions, including executive orders or legislation. The review employed an inductive qualitative content analysis of publicly available documents, including state MPA reports, progress summaries, action plans, planning toolkits, and stakeholder engagement materials.

Each document was systematically coded to identify recurring themes related to age-friendly domains, stakeholder inclusion, implementation strategies, and accountability mechanisms. A matrix comparison approach was then used to compare the scope and structure of state initiatives across geographic, political, and policy contexts. Supplementary insights were gained through informal interviews and correspondence with state leaders, aging advocates, and coalition representatives to validate document findings and clarify ambiguities.

Inclusion criteria focused on states with AARP Age-Friendly designations, MPAs in development or implementation, or relevant legislative/executive activity. States were classified according to the status of their MPA activities. Using a qualitative document review methodology, the team applied thematic coding to identify commonalities across state approaches. Particular attention was paid to how states structured implementation plans, set benchmarks, and integrated stakeholder voices (Berkowsky et al., 2024). The materials analyzed included state action plans and annual reports, MPA documentation, guides, newsletters, and web content, as well as conference presentations, media reports, and correspondence with state-level stakeholders.

**Figure 1:**
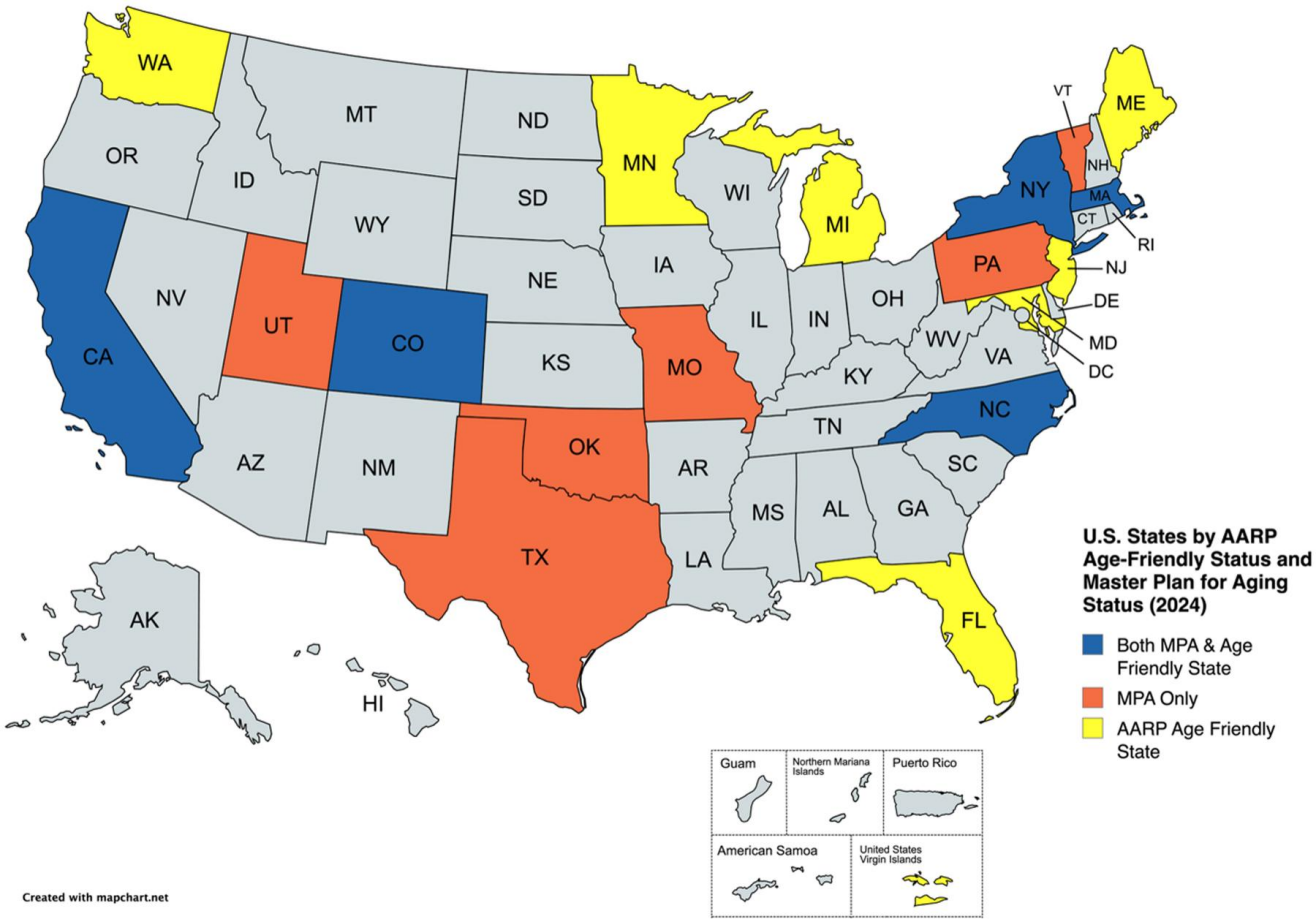
U.S. states by age-friendly state status and MPA status/note. This map is based on 2024 data. MPA = master plan on aging.

States were selected for review based on either having an AARP Age-Friendly State Designation or documented Multisector Plan on Aging (MPA) activity, including legislation, executive orders, or implementation plans. At the time of our analysis in 2024, 18 states and one territory met these criteria. Twelve states had AARP Age-Friendly State Designations, eight states had an MPA in the development or implementation phases, and four states had an MPA with Legislation or an Executive Order in place. Recent surveys, however, suggest that the landscape is shifting rapidly: 16 states now have formal authorization to initiate an MPA, with an additional 22 exploring or planning efforts (Rauscher et al., 2024). Yet, only eight states have fully developed and are actively implementing MPAs (Berkowsky et al., 2024). These updated figures underscore the fluidity of the policy environment and the importance of ongoing tracking. See Table 1 for a list of the states selected for review, in addition to Age-Friendly State status, enrollment year, and status of Multisector Plan on Aging. See Figure 1 for a visual representation of states that have an MPA, states with a state AARP Age-Friendly designation, states with both an MPA and AARP state designation, and states that were not selected based on the criteria for this study.

**Table 1:** States selected for review by age-friendly state and MPA status.

State	AARP age-friendly state designation	AARP enrollment	MPA	MPA status
California	Yes	2021	Yes	Developed/Implementing
Colorado	Yes	2018	Yes	Developed/Implementing
Florida	Yes	2019	No	N/A
Maine	Yes	2019	No	N/A
Maryland	No	N/A	Yes	Legislation/Executive Order
Massachusetts	Yes	2018	Yes	Developed/Implementing
Michigan	Yes	2019	No	N/A
Minnesota	Yes	2022	No	N/A
Missouri	No	N/A	Yes	Legislation/Executive Order
New Jersey	Yes	2021	No	N/A
New York	Yes	2017	Yes	Legislation/Executive Order
North Carolina	Yes	2023	Yes	Legislation/Executive Order
Oklahoma	No	N/A	Yes	Developed/Implementing
Pennsylvania	No	N/A	Yes	Developed/Implementing
Texas	No	N/A	Yes	Developed/Implementing
US Virgin Islands	Yes	2019	No	N/A
Utah	No	N/A	Yes	Developed/Implementing
Vermont	No	N/A	Yes	Developed/Implementing
Washington	Yes	2024	No	N/A

*Note.* This table is based on 2024 data. MPA = master plan on aging; N/A = not applicable.

### Pathways that only states can forge

States have adopted a variety of strategies, each yielding tangible outcomes that highlight the potential of state-led interventions to influence aging policy and practice across domains. The following section outlines key contributions to long-term improvements in aging policy and practice that emerged from the environmental scan conducted. The five main themes identified include (1) financing and coverage infrastructure, (2) housing and land use reform, (3) workforce and caregiver supports, (4) governance reforms, and (5) stakeholder engagement.

### Financing and coverage infrastructure

Across states, age-friendly policy development increasingly includes reforms to public financing mechanisms, insurance eligibility, and income supports that shape access to long-term services and supports (LTSS). Rather than isolated program expansions, these efforts reflect attempts to recalibrate the fiscal architecture that determines who can afford care and under what conditions. Several states have expanded eligibility for publicly financed health coverage or modernized income-based supports to reduce gaps among middle- and lower-income older adults. For example, California expanded Medi-Cal eligibility to adults aged 50 and older regardless of immigration status, extending coverage to an estimated 235,000 individuals (Office of Governor Gavin Newsom, 2022). Pennsylvania updated its Property Tax/Rent Rebate Program by increasing maximum rebates, expanding income thresholds, and indexing eligibility to inflation (Commonwealth of Pennsylvania, Department of Revenue, 2024). Oklahoma enacted the Caring for Caregivers Act, establishing a refundable tax credit for eligible unpaid family caregivers (AARP Oklahoma, 2023; Burkhead, 2024).

Although these policies operate through different instruments (insurance expansion, tax relief, and income stabilization), they share a common objective: reducing structural financial barriers to care. Collectively, they illustrate how states are using coverage policy and fiscal tools to address affordability and access as foundational components of age-friendly infrastructure.

### Housing and land use reform

Housing policy has emerged as a central structural domain of state-level aging reform. Across states, land use regulations and zoning laws are being revisited to expand housing flexibility, increase supply, and reduce regulatory barriers that limit aging in place. Accessory dwelling unit (ADU) reforms represent a recurring strategy. Massachusetts authorized ADUs “by right,” reducing zoning barriers and expanding opportunities for multigenerational living (AARP, 2024; Massachusetts Executive Office of Elder Affairs, 2024). Washington State enacted House Bill EHB 1337, requiring cities and counties to allow ADUs in urban growth areas and limiting restrictive zoning practices (Washington State Legislature, 2023). In addition, Florida created a Senior Housing Liaison position within the Department of Elder Affairs to coordinate housing innovation and interagency efforts (Florida Department of Elder Affairs, 2023a). These reforms primarily alter the regulatory framework governing housing markets rather than directly delivering housing services. As such, they represent structural interventions designed to align land use systems with demographic aging trends.

### Workforce and caregiver supports

Workforce stabilization and caregiver support constitute a core cross-cutting policy domain. States are using labor policy, targeted wage investments, and caregiver-focused supports to address shortages in direct care occupations and reduce strain on unpaid caregivers. Michigan’s FY2024 budget allocated $140 million to increase wages for direct care workers in home- and community-based and residential settings (Office of Governor Gretchen Whitmer, 2023). Colorado implemented Paid Family and Medical Leave Insurance (FAMLI), providing up to 12 weeks of paid leave for caregiving and medical needs (Colorado Department of Labor and Employment, 2025; Rauscher et al., 2024), alongside Career Advance Colorado, which expands workforce training pipelines in high-demand care sectors (Colorado Office of Economic Development and International Trade, n.d.).

Some states have supplemented statutory reforms with programmatic supports. Partnerships with Trualta in New York and Washington State provide digital caregiver education and peer support at no cost to unpaid caregivers (New York State Office for the Aging [NYSOFA], 2022; Washington State Department of Social and Health Services, n.d.). Evidence suggests that structured caregiver education platforms can improve self-efficacy and reduce burden (Rodriguez et al., 2021). Massachusetts also launched an Age-Friendly Employer Certification Program to encourage age-inclusive workplace practices (Massachusetts Executive Office of Aging & Independence, 2022). Together, these strategies demonstrate how workforce capacity and caregiver resilience are being addressed through integrated labor, fiscal, and programmatic tools.

### Governance reforms

A distinct set of state actions involves institutional restructuring and formal governance adjustments that elevate aging within administrative systems. Massachusetts filed legislation to rename the Executive Office of Elder Affairs as the Executive Office of Aging & Independence, signaling a broader mandate and reframing aging policy within an autonomy-oriented framework (Massachusetts Executive Office of Health and Human Services, 2024). Missouri’s Silver Haired Legislature provides a formally elected body of residents aged 60 and older that identifies policy priorities and advances model legislation (Missouri Department of Health & Senior Services, n.d.). Colorado’s Lifelong Colorado initiative has supported more than 100 municipalities in adopting age-friendly community plans, aligning local and state planning efforts (Rauscher et al., 2024). These governance reforms alter institutional architecture and policy coordination mechanisms, embedding aging considerations within broader systems of state administration.

### Stakeholder engagement

States are also advancing age-friendly goals through structured stakeholder engagement and cross-sector collaboration mechanisms. Florida’s Hope Florida initiative integrates government, nonprofit, private, and faith-based organizations to coordinate support for older adults (Florida Department of Elder Affairs, 2023b). Missouri’s Communities for All Ages Recognition Program provides technical assistance and a tiered designation system to municipalities working to strengthen supports for older residents (Mid-America Regional Council, n.d.). Utah developed public education materials to raise awareness about fraud targeting older adults (Utah Insurance Department, n.d.).

Universities also play a role in engagement infrastructure. The University of Maryland, Baltimore operates the Age-Friendly University Geri-ED platform (University of Maryland, Baltimore, n.d.), and the University of Massachusetts system formally endorsed the Age-Friendly University principles across its campuses (Office of Communications, University of Massachusetts, 2019). These initiatives function as connective infrastructure, linking policy objectives with community implementation and reinforcing civic participation within age-friendly ecosystems.

Across these five domains – financing infrastructure, housing reform, workforce support, governance restructuring, and stakeholder engagement – states are employing varied but convergent policy tools to respond to demographic aging. Organizing these actions by policy function rather than geography clarifies the structural mechanisms through which states are operationalizing age-friendly systems change. See Tables 2 and 3 for examples of state strategies across the five domains of Age-Friendly systems change.

**Table 2:** Domains and illustrative state strategies in age-friendly systems development.

Domain	Policy instruments	Illustrative examples	Structural function
Financing and coverage infrastructure	Medicaid eligibility expansion; property tax and rent relief modernization; refundable caregiver tax credits	California Medi-Cal expansion (adults aged 50+ regardless of immigration status); Pennsylvania property tax/rent rebate update; Oklahoma caring for Caregivers Act	Reduces financial barriers to care; stabilizes housing affordability; redistributes caregiving costs
Housing and land use reform	ADU authorization “by right”; statewide zoning preemption; dedicated senior housing coordination	Massachusetts ADU reform; Washington EHB 1337; Florida Senior Housing Liaison	Expands adaptable housing supply; reduces zoning constraints; aligns land use systems with demographic aging
Workforce and caregiver supports	Direct care wage investments; paid family leave; workforce training pipelines; caregiver education platforms; age-inclusive employer initiatives	Michigan direct care wage allocation; Colorado FAMLI and Career Advance Colorado; New York and Washington Trualta partnerships; Massachusetts age-friendly employer program	Stabilizes LTSS labor supply; mitigates caregiver income loss; strengthens workforce and caregiver capacity
Governance reforms	Agency restructuring/renaming; formal advisory or representative bodies; state–local planning alignment initiatives	Massachusetts Executive Office of Aging & Independence proposal; Missouri Silver Haired Legislature; Colorado Lifelong Colorado	Institutionalizes aging policy leadership; formalizes older adult representation; integrates municipal and state planning
Stakeholder engagement	Cross-sector coordination initiatives; municipal recognition programs; public education campaigns; university partnerships	Florida Hope Florida; Missouri Communities for All Ages; Utah fraud prevention campaign; University of Maryland Geri-ED; University of Massachusetts Age-Friendly University designation	Strengthens civic infrastructure; incentivizes local implementation; expands public awareness and education

*Note.* Illustrative examples are drawn from state legislative documents, administrative reports, and program descriptions cited in the text. ADU = accessory dwelling unit; FAMLI = paid family and medical leave insurance; EHB = Engrossed House Bill; LTSS = long-term services and support.

**Table 3:** Illustrative state strategies across five domains of age-friendly systems change.

Policy domain	Policy mechanism	State example(s)	System-level function
Financing and coverage infrastructure	Medicaid eligibility expansion	California Medi-Cal expansion to adults aged 50+ regardless of immigration status	Expands insurance coverage and reduces access inequities among low-income older adults
	Property tax and rent relief modernization	Pennsylvania Property Tax/Rent Rebate	Stabilizes housing affordability
	Refundable caregiver tax credit	Oklahoma Caring for Caregivers Act	Offsets out-of-pocket caregiving costs
Housing and land use reform	ADU legalization “by right”	Massachusetts ADU reform	Increases housing flexibility, supports multigenerational living, enables aging in place
	Statewide ADU authorization in urban growth areas	Washington State EHB 1337	Reduces local zoning barriers and expands adaptable housing supply
	Senior Housing Liaison position	Florida Department of Elder Affairs	Institutionalizes cross-agency coordination for age-supportive housing development
Workforce and caregiver supports	Direct care wage investment	Michigan FY2024 $140M allocation	Improves retention and stabilizes LTSS labor supply
	Paid Family and Medical Leave	Colorado FAMLI	Reduces income disruption during caregiving and medical leave
	Workforce training expansion	Career Advance Colorado	Expands pipeline into high-demand health and caregiving occupations
	Digital caregiver education partnerships	Trualta in New York and Washington State	Builds caregiver skills and reduces burden at scale
	Age-Friendly Employer Certification	Massachusetts Age-Friendly Employer Program	Encourages labor force participation and age-inclusive workplace practices
Governance reforms	Agency restructuring/renaming	Massachusetts Executive Office of Aging & Independence	Expands mandate and reframes aging policy within broader autonomy framework
	Institutionalized older adult advisory body	Missouri Silver Haired Legislature	Formalizes representation of older residents in policymaking
	State–local planning alignment initiative	Colorado Lifelong Colorado	Coordinates municipal and state-level age-friendly planning
Stakeholder engagement	Cross-sector service coordination	Florida Hope Florida	Integrates public, nonprofit, faith-based, and private partners
	Municipal recognition; technical assistance	Missouri Communities for All Ages	Incentivizes local policy adoption and implementation
	Public fraud prevention education	Utah Insurance Department videos	Enhances protection awareness among older adults
	University engagement in aging networks	University of Maryland Geri-ED; UMass Age-Friendly University designation	Connects higher education to aging policy ecosystems

*Note.* ADU = accessory dwelling unit; FAMLI = Paid Family and Medical Leave Insurance; EHB = Engrossed House Bill; LTSS = long-term services and supports.

## State pathways as infrastructure for sustaining age-friendly ecosystems

Findings from this analysis suggest that state involvement plays a critical role in transforming age-friendly efforts from discrete local initiatives into sustained, system-level change. Across states, successful age-friendly strategies are distinguished not by any single policy or program, but by the presence of governance pathways that align local innovation, health care transformation, and broader inclusion-oriented systems change related to age and disability. These pathways create the infrastructure necessary to embed age-friendly principles into the routine functions of government and public systems.

States that have made meaningful progress share several interrelated characteristics. First, they demonstrate strong multisector coordination that bridges aging services, health care, housing, transportation, labor, and public health. This alignment enables states to address aging not as a siloed issue, but as a cross-cutting policy concern that intersects with chronic disease management, workforce development, and community design. Participation in national collaboratives and partnerships with academic and public health institutions further supports this coordination by providing shared frameworks, technical assistance, and opportunities for peer learning (Greenfield & Pestine-Stevens, 2022; Rauscher et al., 2024). Second, effective states move beyond symbolic commitments by institutionalizing accountability. Cross-agency governance structures, shared performance metrics, and public-facing dashboards allow aging-related goals to be tracked, monitored, and integrated across departmental agendas. These mechanisms increase transparency and durability, reducing reliance on individual champions and insulating age-friendly efforts from leadership turnover. States such as Massachusetts, which have embedded age-friendly principles into legislation, illustrate how codified accountability can accelerate diffusion and sustain momentum, compared to states that rely primarily on executive orders or voluntary coalitions (Coyle et al., 2022; Graham, 2024).

Third, leadership and stakeholder engagement emerge as foundational elements of infrastructure-building. As pointed out by Greenfield et al., the development of age-friendly initiatives requires coordinated efforts that involve diverse stakeholders across various sectors (Greenfield et al., 2022; Colorado Office of Economic Development and International Trade, n.d.). States that meaningfully involve older adults, caregivers, and disability advocates in planning and implementation are better positioned to address inequities and service gaps. These participatory processes ensure that state strategies reflect lived experience and reinforce the alignment between aging and disability policy, including Olmstead obligations and home- and community-based services planning (Berkowsky et al., 2024). Previous studies surrounding age-friendly communities have also emphasized the importance of strong leadership and governance (Forsyth & Lyu, 2024; Greenfield & Pope, 2025; Menec & Brown, 2022; Torku et al., 2021). Academic partnerships further strengthen this work by supporting community needs assessments, evaluation, and the generation of comparable data across jurisdictions (Pope et al., 2025).

Taken together, these pathways allow states to serve as connective tissue between local age-friendly initiatives, health care delivery systems, and broader efforts to advance inclusion across the life course. Rather than operating as standalone programs, age-friendly principles become embedded within budgeting processes, workforce strategies, land-use policy, and emergency preparedness planning. This shift reflects a deeper cultural transformation within government, one that reframes aging as a universal experience rather than a niche population issue (Greenfield & Pope, 2025).

States that blend top–down authority with bottom–up engagement appear particularly well positioned to build durable age-friendly ecosystems. Legislative and administrative actions establish an enabling environment by signaling political commitment, allocating resources, and mandating coordination, while community-led efforts foster legitimacy and responsiveness. Examples from California, New York, and Washington demonstrate how this hybrid approach can generate early wins such as Medicaid expansions, zoning reforms, digital tools, and caregiver training platforms that serve as building blocks for longer-term systems change (Greenfield & Pope, 2025; Rauscher et al., 2024).

Finally, emerging federal momentum, including proposed legislation such as the Strategic Plan for Aging Act (S.3827), underscores the opportunity to further align state and federal aging policy. Federal support for state planning and implementation could accelerate the diffusion of best practices and reinforce states’ role as intermediaries between national priorities and local action. Positioned within this evolving policy landscape, state-level age-friendly pathways have the capacity to sustain, scale, and normalize age-friendly principles as a core feature of public systems rather than a time-limited initiative.

## Barriers and structural constraints

Despite progress, states face several barriers and challenges in implementing age-friendly practices at the state level. Specifically, research within the age-friendly movement has highlighted disparities in the inclusion of diverse aging populations (Buffel & Phillipson, 2018; Greenfield, 2018; Oswald & Cooper, 2024). Building on this critique, this scan suggests that the most persistent constraints are structural—rooted in governance fragmentation, uneven implementation capacity, and fiscal and political volatility that can limit durability over time. Age-friendly work requires coordinated action across Medicaid agencies, housing and community development, transportation, labor, public health, emergency management, and aging services. Yet state administrative structures frequently mirror sector silos, with separate statutory mandates, contracting rules, performance metrics, and funding timelines. Even when MPAs or Age-Friendly State plans articulate cross-cutting priorities, implementation can stall without a designated cross-agency “backbone” entity, shared performance indicators, and decision rights over budgets and procurement. In practice, many state initiatives rely on interagency goodwill and short-term convening rather than formalized governance arrangements that can survive leadership transitions. **When it comes to political turnover,** many plans emphasize “quick wins” to demonstrate momentum, but multiyear systems change typically requires sustained appropriations, braided funding strategies, and administrative authority that extends beyond a single gubernatorial term. States that rely primarily on voluntary coalitions or executive-led initiatives may face abrupt shifts in priorities, staffing, and reporting expectations. This volatility can weaken local confidence, reduce participation, and limit the ability to build a mature implementation infrastructure (e.g., data systems, workforce pipelines, technical assistance) that is essential for scaling. Although MPAs emphasize data-informed planning, states vary widely in analytic capacity and in the availability of interoperable data systems that connect health, housing, transportation, and LTSS. Many plans rely on process measures (e.g., number of stakeholders engaged, toolkits developed) rather than outcome and equity indicators (e.g., reductions in unmet LTSS need, caregiver burden, falls, avoidable hospitalizations, housing cost burden, transportation access). In addition, data gaps can obscure inequities among rural residents, immigrants, people with disabilities, and LGBTQ+ older adults, limiting the ability to design targeted strategies and to monitor progress toward distributive and procedural justice (Greenfield, 2018; Oswald & Cooper, 2024). Future research should examine the long-term effects of age-friendly state initiatives on equity, service delivery, civic engagement, and the durability of structural policy changes (Black & Oh, 2022). Longitudinal and mixed-methods approaches will be particularly valuable for documenting outcomes over time and validating emerging best practices (Pope et al., 2025).

## Conclusion

Overall, this review underscores that successful age-friendly states intentionally embed aging considerations into core governance structures, leverage multisector partnerships, and use data to shape priorities and track progress. These efforts align with broader goals related to health equity, economic development, transportation, and housing (AARP, 2023). At their best, Age-Friendly State initiatives and MPAs function less as discrete “aging programs” and more as state-level operating systems—reframing aging as a cross-cutting policy lens that shapes how mainstream agencies plan, fund, and measure their work across the life course. Looking ahead, states can strengthen progress by investing in sustainable infrastructure and workforce development, expanding age-friendly planning capacity across agencies, and establishing ongoing mechanisms for community engagement. Supporting local innovation through flexible funding and technical assistance will further enable communities to tailor strategies to their unique needs (Scharlach, 2017).

The environmental scan suggests that states serve as a linchpin in age-friendly ecosystems in three interlocking ways. First, they create authorizing environments through statutes, executive actions, and administrative policy changes that enable local age-friendly implementation at scale (e.g., statewide housing reforms, Medicaid and caregiver supports). Second, states provide coordination infrastructure by aligning agencies, standardizing frameworks, and convening partners across public, private, and community sectors—activities that are difficult to replicate at the municipal level. Third, states can establish accountability and continuity, including shared metrics, public reporting, and durable funding mechanisms that reduce reliance on individual champions and support long-term systems change.

To promote consistency and accelerate the diffusion of best practices, developing a national framework or shared standards for age-friendly state planning, supported by technical assistance, capacity-building resources, and a centralized platform for sharing tools, metrics, and lessons learned can solidify Age-Friendly state planning as a system of change. Such a framework would help states benchmark progress, identify gaps, and promote collaboration.

Ultimately, sustaining the age-friendly movement will require strong leadership, cross-sector alignment, and a shared commitment to advancing equitable, inclusive aging (Greenfield & Pope, 2025). State governments are uniquely positioned to accelerate this work and to institutionalize changes that support aging with dignity, autonomy, and well-being for generations to come.

## Data Availability

This article does not report data and therefore the pre-registration and data availability requirements are not applicable.

## References

[gnag063-B1] AARP. (n.d.). *AARP network of age-friendly states and communities.* Retrieved September 30, 2025, from https://www.aarp.org/livable-communities/network-age-friendly-communities/

[gnag063-B3] AARP Massachusetts. (2024, June 6). *Battling loneliness by being a good neighbor*. AARP Massachusetts. https://states.aarp.org/massachusetts/battling-loneliness-by-being-a-good-neighbor

[gnag063-B4] AARP Oklahoma. (2023, June 12). *Oklahoma passes tax credit for family caregivers*. AARP Oklahoma. https://states.aarp.org/oklahoma/oklahoma-passes-tax-credit-for-family-caregivers

[gnag063-B5] AARP. (2023). *Valuing the invaluable 2023 update: Strengthening supports for family caregivers*. AARP Public Policy Institute. https://www.aarp.org/ppi/info-2023/valuing-the-invaluable-2023.html

[gnag063-B7] Berkowsky R. W. , Adams B., Villa R., Glenn T. I. (2024). S. 3827 and a systematic review of state-level multisector plans for aging: How incorporating volunteer caregiving can mitigate service gaps. Inquiry: A Journal of Medical Care Organization, Provision and Financing, 61, 469580241285166. 00469580241285166. 10.1177/00469580241285166 39302738 PMC11526310

[gnag063-B8] Black K. , Oh P. (2022). Assessing age-friendly community progress: What have we learned? The Gerontologist, 62, 6–17. 10.1093/geront/gnab051 33870431

[gnag063-B9] Buffel T. , Phillipson C. (2018). A manifesto for the age-friendly movement: Developing a new urban agenda. Journal of Aging & Social Policy, 30, 173–192. 10.1080/08959420.2018.1430414 29364777

[gnag063-B10] Burkhead K. (2024, January 3). *Oklahoma’s caring for caregivers act offers new tax credits for unpaid family caregivers*. KTUL. https://ktul.com/news/local/oklahomas-caring-for-caregivers-act-offers-new-tax-credits-for-unpaid-family-caregivers-medical-equipment-ramps-technology-home-health-services-ok-dementia-veterans-aarp-diagnosis-emotional-burden

[gnag063-B1300] Colorado Office of Economic Development and International Trade. (n.d.). Skill Advance Colorado job training grant. State of Colorado. Retrieved May 7, 2026, from https://oedit.colorado.gov/skill-advance-colorado-job-training-grant

[gnag063-B13] *Colorado Family and Medical Leave Insurance (FAMLI) program*. Retrieved July 10, 2025, from https://famli.colorado.gov/

[gnag063-B14] Commonwealth of Pennsylvania, Department of Revenue. (2024, June 28). *In Centre County, Governor Shapiro and Secretary Kavulich visit senior center, announce 2023 property tax/rent rebate checks will go out starting July 1, and remind eligible Pennsylvanians to apply*. https://www.pa.gov/agencies/revenue/newsroom/in-centre-county-governor-shapiro-and-secretary-kavulich-visit-senior-center-announce-2023-property-taxrent-rebate-checks-will-go-out-starting-july-1-and-remind-eligible-pennsylvanians-to-apply

[gnag063-B15] Coyle C. E. , Gleason S. R., Mutchler J. E. (2022). Spillover benefits and achieving sustainability of age-friendly communities. The Gerontologist, 62, 29–35. 10.1093/geront/gnab152 33982096

[gnag063-B17] Florida Department of Elder Affairs. (2023a). *2023 year in review.* https://elderaffairs.org/wp-content/uploads/2023-Year-in-Review_WEB.pdf

[gnag063-B18] Florida Department of Elder Affairs. (2023b, December 28). *Elder affairs continues to advance innovations and enhance critical programming for Florida’s cherished seniors and their families.* https://elderaffairs.org/elder-affairs-continues-to-advance-innovations-and-enhance-critical-programming-for-floridas-cherished-seniors-and-their-families/

[gnag063-B20] Forsyth A. , & Lyu Y. (2024). Making Communities Age-Friendly: Lessons From Implemented Programs. Journal of Planning Literature, 39, 3–24. 10.1177/08854122231160796

[gnag063-B21] Graham C. (2024). State multisector plans for aging can promote a more coordinated “ecosystem” for older adults. *Generations Today*, 489, 1–6.

[gnag063-B22] Graham C. , Hoffmaster A. (2022). *Developing a multisector plan for aging*. Center for Health Care Strategies. June.

[gnag063-B23] Greenfield E. A. (2018). Age-Friendly Initiatives, Social Inequalities, and Spatial Justice. *The Hastings Center Report*, 48 Suppl 3, S41–S45. 10.1002/hast.912 30311225 30311225

[gnag063-B24] Greenfield E. A. , Black K., Oh P., Pestine-Stevens A. (2022). Theories of community collaboration to advance age-friendly community change. The Gerontologist, 62, 36–45. 10.1093/geront/gnab153 34528063

[gnag063-B25] Greenfield E. A. , Pestine-Stevens A. (2022) Evaluating age-friendly community initiatives from a social network perspective. Innovation in Aging, 6, 72–72. 10.1093/geroni/igac059.289

[gnag063-B26] Greenfield E. A. , Pope N. E. (2025). “It made me change the way I do business”: Outcomes from age-friendly community initiatives as systems change. The Gerontologist, 65, gnae149. 10.1093/geront/gnae149 PMC11638776 39450442

[gnag063-B30] Massachusetts Executive Office of Aging & Independence. (2022, April 29). *Massachusetts becomes first state certified as an age-friendly employer.* https://www.mass.gov/news/massachusetts-becomes-first-state-certified-as-an-age-friendly-employer

[gnag063-B31] Massachusetts Executive Office of Elder Affairs. (2024). *Massachusetts becomes first state certified as an age-friendly employer.* Retrieved July 10, 2025, from https://www.mass.gov/news/massachusetts-becomes-first-state-certified-as-an-age-friendly-employer

[gnag063-B32] Massachusetts Executive Office of Health and Human Services (2024, May 28). *Governor Healey files legislation to rename the Executive Office of Elder Affairs to Executive Office of Aging & Independence*. https://www.mass.gov/news/governor-healey-files-legislation-to-rename-the-executive-office-of-elder-affairs-to-executive-office-of-aging-independence

[gnag063-B33] Menec V. H. , Brown C. (2022). Facilitators and barriers to becoming age-friendly: A review. Journal of Aging & Social Policy, 34, 175–197. 10.1080/08959420.2018.1528116 30321112

[gnag063-B34] Mid-America Regional Council. (n.d). *Communities for all ages*. Retrieved November 20, 2025, from https://www.marc.org/aging/communities-all-ages

[gnag063-B35] Missouri Department of Health & Senior Services. (n.d). *Silver Haired Legislature.* https://health.mo.gov/seniors/silverhaired/

[gnag063-B37] New York State Office for the Aging. (2022). *NYSOFA, AgingNY, and Trualta provide free web-based support platform to all family caregivers in NYS.* Retrieved July 10, 2025, from https://aging.ny.gov/news/nysofa-agingny-and-trualta-provide-free-web-based-support-platform-all-family-caregivers-nys

[gnag063-B38] Office of Communications, University of Massachusetts. (2019, September 25). *University of Massachusetts becomes first university system to join Age-Friendly University Global Network.* https://www.massachusetts.edu/news/university-massachusetts-becomes-first-university-system-join-age-friendly-university-global

[gnag063-B39] Office of Governor Gavin Newsom. (2022, April 29). *California expands Medi-Cal to all eligible adults 50 years of age and older.* https://www.gov.ca.gov/2022/04/29/california-expands-medi-cal-to-all-eligible-adults-50-years-of-age-and-older/

[gnag063-B40] Office of Governor Gretchen Whitmer. (2023). *Whitmer applauds passage of Make it in Michigan budget.* Retrieved July 10, 2025, from https://www.michigan.gov/whitmer/news/press-releases/2023/06/28/whitmer-applauds-passage-of-make-it-in-michigan-budget#:∼:text=The%20budget%20makes%20vital%20investments, nursing%20home%20services%20and%20supports

[gnag063-B41] Oswald A. G. , Cooper L. (2024). Addressing equity and justice in age-friendly communities: Considerations for LGBTQ+ older adults of color. The Gerontologist, 64, gnae050. 10.1093/geront/gnae050 38767047

[gnag063-B42] Pope N. E. , Greenfield E. A., Keyes L., & Russell E. (2025). A Review of Public Sector Engagement in Age-Friendly Community Initiatives. Journal of Aging & Social Policy, 37, 187–215. 10.1080/08959420.2024.2376934 39158025

[gnag063-B43] Rauscher E. , Phan M., Graham C., Somers S. (2024). *The state of multisector plans for aging in 2024*. Center for Health Care Strategies. https://www.chcs.org/resource/the-state-of-multisector-plans-for-aging-in-2024/

[gnag063-B44] Rodriguez K. , Fugard M., Amini S., Smith G., Marasco D., Shatzer J., Guerrero M., Garvan C., Davis J., Price C. (2021). Caregiver response to an online dementia and caregiver wellness education platform. Journal of Alzheimer’s Disease Reports, 5, 433–442. 10.3233/ADR-210294 PMC8293668 34368629

[gnag063-B46] Scharlach A. E. (2017). Creating aging-friendly communities in the United States. Ageing International, 42, 20–28. 10.1007/s12126-017-9270-1

[gnag063-B50] Torku A. , Chan A. P. C., Yung E. H. K. (2021). Age-friendly cities and communities: A review and future directions. Ageing and Society, 41, 2242–2279. 10.1017/S0144686X20000239

[gnag063-B51] University of Maryland, Baltimore. (n.d). *Geri-Ed: A platform for educational programs in geriatrics and gerontology.* https://www.umaryland.edu/gerontology/education-and-training/school-of-graduate-studies/geri-ed/

[gnag063-B52] Utah Insurance Department. (n.d). *Fraud multimedia.* https://insurance.utah.gov/fraud/multimedia/

[gnag063-B53] Washington State Department of Social and Health Services. (n.d). *WA caregiver journey*. Retrieved July 10, 2025, from https://www.wacaregivingjourney.com/

[gnag063-B54] Washington State Legislature (2023, March 16). *House bill report: EHB 1337* [PDF]. https://lawfilesext.leg.wa.gov/biennium/2023-24/Pdf/Bill%20Reports/House/1337.E%20HBR%20PL%2023.pdf

[gnag063-B55] World Health Organization. (2007). Global age-friendly cities: A guide. World Health Organization.

